# Underwater origami method for the closure of large colorectal endoscopic submucosal dissection defects: A strategy to eliminate submucosal dead space

**DOI:** 10.1055/a-2938-1547

**Published:** 2026-08-31

**Authors:** Xiangyue Qi, Hongbo Wu, Zhiwei Tao, Tianqi Xu, Lu Li, Bin Qin

**Affiliations:** 1 Department of Gastroenterology 117799 Xi'an Jiaotong University Second Affiliated HospitalXi'anShaanxiChina

Delayed adverse events (DAEs), such as bleeding and perforation following colorectal endoscopic submucosal dissection (ESD), often lead to severe clinical consequences. Prophylactic defect closure is strongly advocated to reduce DAE risks, particularly in high-risk patients. In this case series, we successfully performed the underwater origami method in two cases of large rectal post-ESD defects ( Video 1 ).

**Video 1** Underwater origami method for the closure of large post-ESD defects in two cases.


**Fig. 1:**
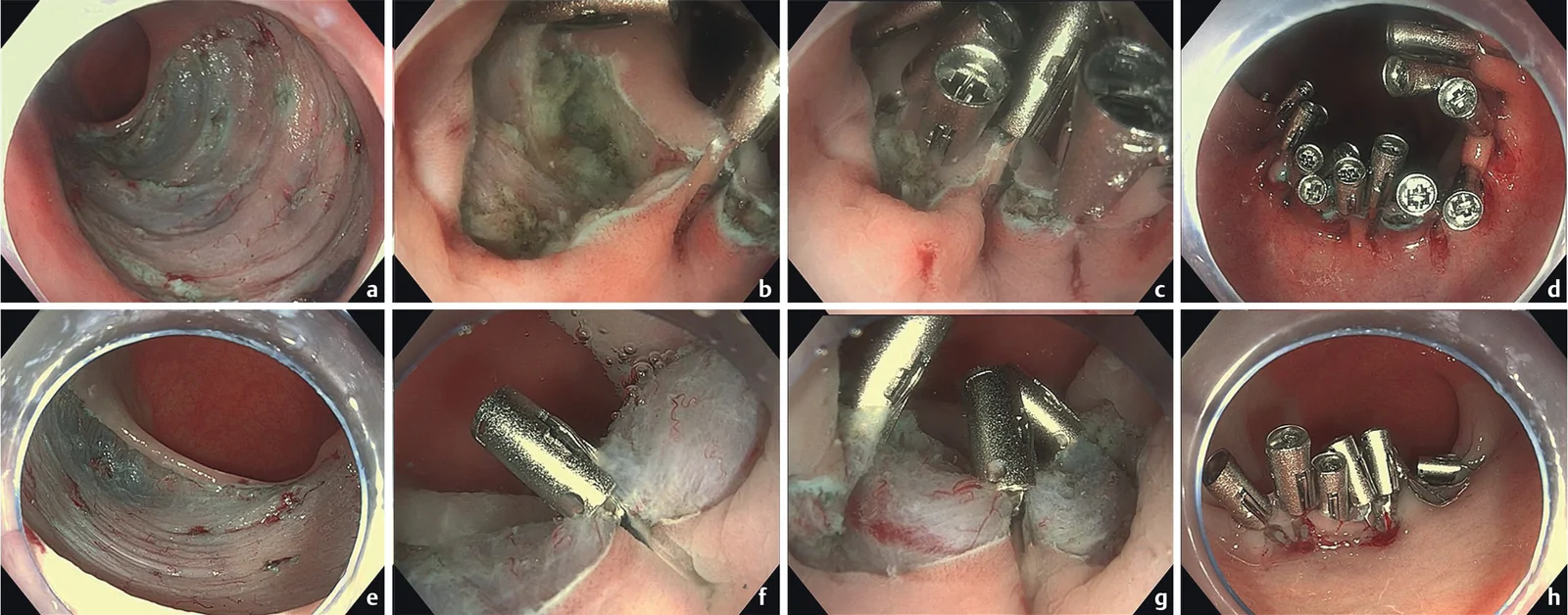
Underwater origami method for the closure of large post-ESD defects. Case1: ( **a** ) Post-ESD mucosal defect. ( **b** ) A submucosal dead space was formed. ( **c** ) The origami method was applied to eliminate the dead space. ( **d** ) Complete closure without residual dead space. Case2: ( **e** ) Post-ESD mucosal defect. ( **f** ) Direct application of the origami method prevented the formation of a submucosal dead space. ( **g** ) Clips were used to anchor the muscularis propria to the marginal mucosa. ( **h** ) Complete closure without dead space.

The first case involved a 70-year-old female who presented with a granular-type laterally spreading tumor (LST-G). She was considered at high risk for post-ESD bleeding due to long-term aspirin therapy for coronary heart disease. Following a complete en bloc resection, a large mucosal defect measuring 4 cm was observed ( Fig. 1a ). Standard closure using conventional hemoclips was precluded by significant luminal tension and air-induced overdistension. We employed an underwater technique, immersing the defect in saline to facilitate mucosal contraction and alleviate tension. Initial underwater closure attempts using through-the-scope clips (TTSCs) resulted in the formation of a submucosal "dead space" (cavity) between the closed mucosal edges and the underlying muscularis propria ( Fig. 1b ). Consequently, we transitioned to the "origami method." By intentionally performing deep grasping to fold the muscularis propria along with the mucosa, both layers were tightly apposed, effectively eliminating the cavity ( Fig. 1c, d ).

In the second case with rectal LST-G, we achieved closure of the 3.5 cm post-ESD defect ( Fig. 1e ) using conventional TTSCs. To prevent cavity formation, we first immersed the defect in saline and then anchored the muscle layer to the marginal mucosa using several clips to fold the tissue. Once the wound area had decreased and tension was sufficiently reduced, we utilized additional hemoclips to achieve complete defect closure without residual dead space ( Fig. 1f–h ).

**Fig. 2:**
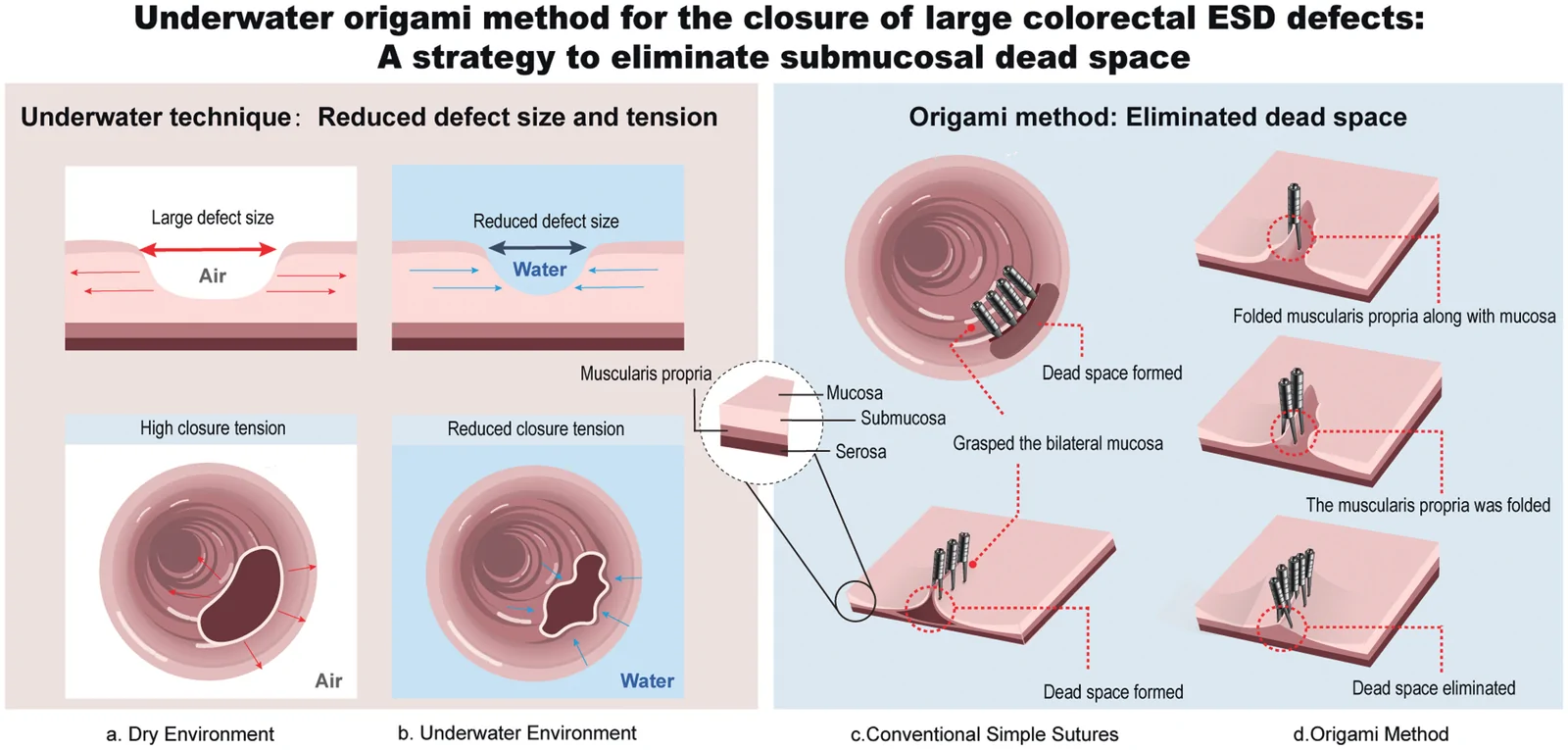
Schematic illustration of underwater origami method. Comparison of defect size and tension in air ( **a** ) versus underwater environment ( **b** ). The underwater technique reduces defect size and tension. Comparison of conventional simple sutures ( **c** ) versus origami method ( **d** ). The origami method eliminates submucosal dead space.

The integration of the underwater technique with the "origami method" offers a strategic advantage for managing large colorectal ESD defects. It overcomes the tension limitations associated with standard clips **(** Fig.2a, b **)** , effectively closing large defects without leaving a submucosal cavity and potentially reducing the risk of DAEs **(** Fig.2c, d **)** . Furthermore, this approach is technically feasible and can be performed using standard single-channel endoscopes by endoscopists trained in routine clip placement.

Endoscopy_UCTN_Code_TTT_1AQ_2AD_3AD

